# Impact of swiping direction on the interaction performance of elderly-oriented smart home interface: EEG and eye-tracking evidence

**DOI:** 10.3389/fpsyg.2023.1089769

**Published:** 2023-02-09

**Authors:** Chengmin Zhou, Ziyan Shi, Ting Huang, Hanxiao Zhao, Jake Kaner

**Affiliations:** ^1^College of Furnishings and Industrial Design, Nanjing Forestry University, Nanjing, Jiangsu, China; ^2^Jiangsu Co-Innovation Center of Efficient Processing and Utilization of Forest Resources, Nanjing Forestry University, Nanjing, Jiangsu, China; ^3^School of Art and Design, Nottingham Trent University, Nottingham, United Kingdom

**Keywords:** swiping direction, interactive performance, interface design, older person design, smart home

## Abstract

**Introduction:**

Smart home technology is increasingly popular, yet not all seniors are receptive and comfortable with it. This situation recognizes that the usability of smart home interfaces is particularly important. Most studies on interface swiping direction demonstrate the advantages of horizontal over vertical swiping, but the findings lack age-based as well as gender-specific judgments.

**Methods:**

In this paper, we use cognitive neural techniques of EEG and eye-tracking, combined with a subjective preference questionnaire, to analyze the preference of older persons for the swiping direction of smart home interfaces in a multimodal manner.

**Results:**

The EEG data showed that swiping direction had a significant effect on potential values (*p* = 0.001). Also, the mean power in the δ and the θ band was enhanced during vertical swiping. Gender had no significant effect on potential values (*p* = 0.085), but the cognitive task was more EEG stimulating for females. The eye-tracking metrics data showed a significant effect of swiping direction on fixation duration (*p* = 0.047) and a non-significant effect on pupil diameter (*p* = 0.576). These results were consistent with the results of the subjective preference questionnaire, both demonstrating a preference for vertical swiping among participants.

**Discussion:**

This paper uses three research tools simultaneously, combining objective perceptions as well as subjective preferences, to make the findings more comprehensive and reliable. Gender differences were also taken into account and differentiated in the data processing. The findings of this paper are different from most previous studies and better reflect the preference of elderly people for swiping directions, which can provide a reference for the future elderly-friendly smart home interface design.

## 1. Introduction

In a very short time, the concept of smart home technology (Harper, 2003) has become increasingly popular and accepted as an intermediate, effective, economical, and sustainable option. The rapid growth of IoT components and devices in recent years has contributed to the expansion of IoT-based solutions such as smart home technology (Herath and Mittal, 2022). Although providing ambient intelligence in domestic settings was the main purpose of this smart house research, there is presently a rising interest in using smart homes to offer a variety of specialized services that specifically target the needs of users (Curumsing et al., 2019; Xiong et al., 2020; Hu et al., 2021). Among them, the elderly-oriented smart home is getting more and more attention (Xiong et al., 2018; Ghorayeb et al., 2021). However, not all older persons are receptive to smart homes. Many seniors show reluctance to the use of new technology (Zajicek, 2004; Kalimullah and Sushmitha, 2017). The way many technology products are designed for use does not consider the cognitive load and emotional preferences of the elderly, which is probably one of the reasons for poor adoption. Therefore, the design, interactive experience, and accessibility of smart home interfaces as a medium of interaction are of particular importance (Li, 2021; Zhang et al., 2022). As a result, many scholars have conducted relevant research in this area. In recent years, in Sharma and Wong (2020), one smart home graphical interface was designed and developed, which ensures comparatively greater ease in operational efforts for the senior residents; In Reddy et al. (2020), a simple words-based interface was considered the most effective for older users; In Yu et al. (2022b), the authors explored the button characteristics (i.e., button size, graphics/text ratio, and icon style) preferred by the elderly, to provide a reference for the key design of the smart home interface.

As the way a user interacts with an interface has such a big impact on their behavioral attitudes, intentions, and outcomes (Choi et al., 2016), it's crucial to investigate practical ways to interact while researching elderly-friendly smart home interfaces (Yu et al., 2022a). It is well known that the boom in touchscreen technology continues to stimulate the emergence of new interaction methods (Lamont and Crawford, 2012; Sedaghatjou and Campbell, 2017), from scrolling and clicking to dragging, flipping, swiping, and zooming, greatly expanding the range of user actions in interfaces. Among these, swipe gestures have been an important topic of research on their characteristics and performance as the main interaction method. Swiping gesture directions are mainly divided into vertical and horizontal swiping. Many studies are comparing these two types of swiping, and most scholars have demonstrated the interaction advantages of horizontal swipe over vertical swipe (or vertical scrolling gestures similar to vertical swipe). In Warr and Chi (2013), the authors found that on mobile browsers, switching interfaces using horizontal swipe gestures had the advantages of faster switching speeds, greater resistance to interference, and no significant effect on error rates compared to vertical scrolling gestures; In Kim et al. (2016), the participants who used swipe gestures for paging were found to be more likely to find relevant documents, click faster, and spend less time on the search results page than those who used scrolling gestures; In Ren et al. (2017), the authors demonstrated a preference for horizontal swiping over vertical swiping for users with high touch level demand, but this difference was not significant for users with low touch level demand; In Jeong and Liu (2017), single-touch motions made horizontally were found to perform better and require less physical effort than movements made vertically or diagonally; In Fierrez et al. (2018), the authors proposed that horizontal swiping is faster than vertical swiping and is independent of device orientation. In their view, horizontal gestures were more discriminative than vertical gestures because data coming from the horizontal direction was more stable. The advantages of horizontal swiping are mainly in the processing of information. Our binocular visual field is horizontal and horizontal eye movements are also required when reading and absorbing textual information Rayner (1998). When swiping horizontally, our eyes follow the visual feedback on the screen horizontally, allowing horizontal eye movements to continue when new information appears, thus making horizontal displays more effective in processing information compared to vertical displays (Deng et al., 2016; Kim et al., 2019). Based on the advantages of horizontal swiping, in Dou and Sundar (2016), the authors suggested that horizontal swiping interaction techniques are also needed on more mobile websites and demonstrate that horizontal swiping can have a positive impact on the behavioral intention to use a website. Furthermore, the two directions of horizontal swiping (left-to-right or right-to-left) have been studied in-depth, suggesting that the direction of the product display can have a suggestive effect on the end consumer's swipe direction, which can further influence more downstream dependent variables such as willingness to pay (Van Kerckhove and Pandelaere, 2018).

However, vertical swiping is not entirely without interaction benefits. In Burnett et al. (2013), the authors demonstrated that vertical swipe vgestures require shorter path lengths and are faster than horizontal swipe gestures, which means that vertical swiping tends to allow people to process information quickly and is better suited to coherent information processing, whereas horizontal swiping is better suited to segmented information processing. In practical interface design, therefore, the choice of swiping direction remains somewhat controversial. In addition, current research findings on swiping direction lack age-based as well as gender-based judgments of differences. Both of these factors have a significant impact on cognitive performance. Firstly, studies for elderly-oriented smart home interfaces must be based on real feedback from older age groups, which does not necessarily correspond to previous experimental findings from general subjects (not age-segregated), as age has a significant effect on task performance (Sjolinder et al., 2003; Pautz et al., 2007; Jiang et al., 2020). Some scholars have suggested that individual user characteristics such as aging affect cognitive performance during human-computer interaction and that the decline in motor skills of the elderly affects the performance of touchscreen gesture operations (Jastrzembski and Charness, 2007; Harada et al., 2013). Many scholars have also demonstrated that young people are more responsive when using touch screens and perform better than older people in gesture operations (Rogers et al., 2000; Tsai et al., 2017). Therefore, it is necessary to conduct a swiping direction preference study for the elderly. Secondly, more scholarly research has demonstrated that gender is an important factor influencing cognitive performance. In Hsieh and Wu (2015), satisfaction with an interface was proved to vary depending on the gender of the user, making it a variable in information-seeking behavior. The authors experimentally demonstrated that women's cognitive performance is significantly better than men's for the user interface currently provided; In Huang and Mou (2021), women were proved to have more usability needs than men by comparing men's and women's cognitive performance for online travel agency websites; In Abbasi et al. (2022), the authors demonstrated that men have better cognitive performance at high noise levels and under high workloads. However, there is little research on gender differences in swiping direction.

With the rapid development of cognitive neuroscience (Seitamaa-Hakkarainen et al., 2016; Chrysikou and Gero, 2020; Slagter and Bouwer, 2021), more and more scholars are considering the use of cognitive neural techniques for evaluating human-computer interaction performance. One of these, the Electroencephalogram (EEG), has proven to be a reliable indicator of spontaneous brain activity (Gevins et al., 1997; Stikic et al., 2011; Herath and de Mel, 2021). In Chen et al. (2017), one electroencephalographic (EEG) method was proposed based on primary band power spectral density (PSD) to assess brain load tasks, and indicated that the channel in the left frontoparietal lobe (Fp1) had the highest correlation with brain load levels; In Kumar and Kumar (2016), the authors demonstrated that the average power in each band can be used for the characterization of cognitive load; In Ismail and Karwowski (2020), the authors summarized the research on EEG in the cognitive domain and indicated that the amplitude of some ERP components (e.g., P3, P2, N1, N2) decreased with increasing workload. increases and decreases. These studies have demonstrated the validity of the EEG for cognitive performance determination. Additionally, eye-tracking is a method that is particularly useful for evaluating how users interact with machines and how much mental effort is required to complete activities (Diego-Mas et al., 2019). Recently, several scholars have focused on improving human-computer interaction through eye movement biometrics for the elderly and disabled, since eye-gaze movement requires relatively little human effort (Madhusanka et al., 2022). And eye-tracking technology is frequently used in the subject research of search behavior (Al-Samarraie et al., 2017; Kim et al., 2018) because the combination of eye-tracking technology to study people's search behavior can provide a reasonable basis for interface design (Wedel, 2018). In recent years, many scholars have used multimodal measures combining EEG and eye-tracking (Kim et al., 2014; Mark et al., 2018; Moon et al., 2019), which compensate for the earlier use of only autonomy reports and behavioral indicators for assessment. This is because none of these earlier methods can detect implicit user responses or provide information about cognitive changes and emotional responses during human-computer interaction (Minnery and Fine, 2009). In this paper, therefore, EEG and eye-tracking techniques were chosen to study the interaction performance of smart home interfaces in order to provide more reliable feedback.

In this paper, we investigate and analyze swiping direction preferences for elderly-oriented smart home interfaces based on EEG data and eye-tracking metrics data, combined with the subjective questionnaire. Distinguished contributions of our work can be summarized as follows:

- Focus on the elderly group to study the preference of swiping direction of the smart home interface. Comparing the interactive performance of vertical swiping and horizontal swiping provides guidance and suggestions for the future elderly-friendly smart home interface design.
- Based on the EEG data and the eye-tracking metrics data combined with the subjective questionnaire, the results are analyzed on two levels: objective perceptions and subjective emotional preferences, making the findings more comprehensive and reliable.
- Gender differences were taken into account and differentiated in the data processing to compare more intuitively the bias in objective data results due to gender differences in the same cognitive task. Although there was no difference in preference for sliding direction, there were differences in the degree of stimulation of EEG and eye-tracking metrics by gender.

The remaining sections of this paper are organized as follows. In Section 2, the experimental methodology and process are described, including the criteria for participant selection, the materials, and apparatus used in the experiment, the specific design and actual procedures of the experiment, and the data acquisition and analysis of the experiment. In Section 3, the results of the EEG data, the results of the eye-tracking metrics data, and the results of the subjective preference questionnaire data were described separately. In Section 4, the results obtained are discussed in-depth, compared with previous research findings, showing the innovative results of this experiment and presenting the limitations of this experiment. Finally, we conclude this study and propose future works in Section 5.

## 2. Materials and methods

Seventeen middle-aged and elderly participants, aged 53–76 years, were recruited for this experiment (nine of them were male and eight were female). The exclusion criteria were: (1) having puffy eyes or droopy eyelids, (2) having had eye surgery, (3) having natural or corrected visual acuity <1.0, (4) having a physical or cognitive impairment, and 5) being left-handed. The age range of male participants was 54 to 72 years, with an average age of 60.22 (SD 5.61); the age range of female participants was 53–76 years, with an average age of 63.25 (SD 8.51). There was no variability by gender sample pair with respect to age (F = 1.192, *p* = 0.292). All participants were known to have experience with smart touch screen devices, but none had experience with smart home devices. In addition, participants were informed of the experimental procedures before the experiment was conducted and all participants signed an informed consent form.

### 2.1. Materials and apparatus

**Figure 1:**
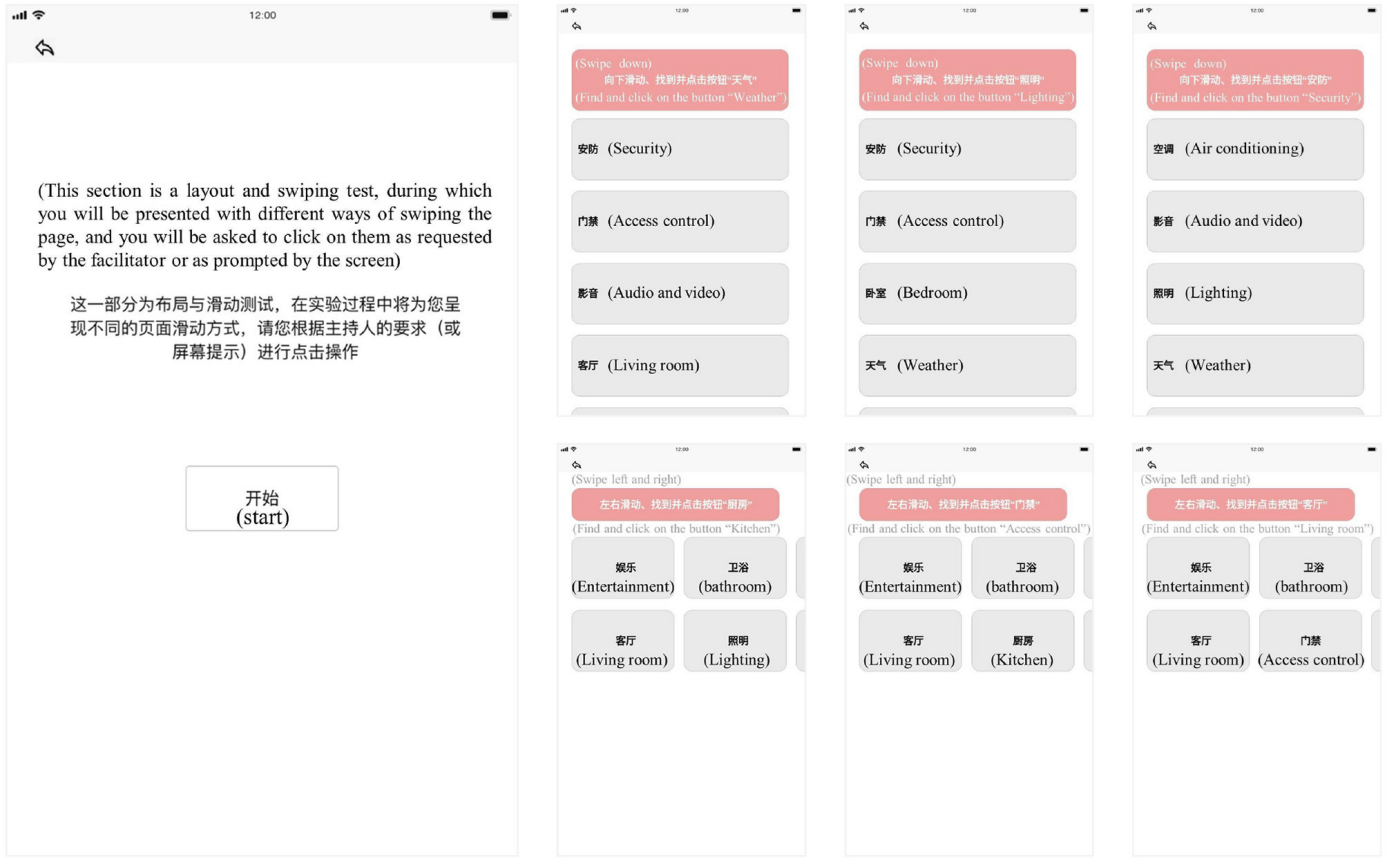
Smart home interface materials for experimental use.

The interface used in the experiment is made by *MockingBot* and presented on the Huawei Honor V6 with an EMUI 10.1 operating system (10.4-inch screen with a resolution of 2000 × 1200 pixels). The stimulus materials for the experiment contained an instruction page interface as well as six test interfaces (three for the vertical swiping layout and three for the horizontal swiping layout), as shown in Figure 1. The experiment employed common smart home functionalities (i.e., security, access control, audio and video, living room, bedroom, lighting, weather, and air conditioning) as the content for text message retrieval.

The experimental data acquisition apparatus is provided by KingFar International Inc and the Beijing Institute of Human Factors Engineering and Technology, including the Ergo LAB human-machine environment synchronization cloud platform, the Semi-Dry wearable wireless EEG measurement system, and the mobile device usability test eye-tracking module (including the mobile terminal test stand and Tobii mobile device test eye-tracking instrument). The Lenovo Savior Y7000 laptop and the Huawei Honor V6 were the other gadgets. Lenovo Savior Y7000 laptop was utilized to use the Ergo LAB human-machine environment synchronization cloud platform, and record experimental data and information about participants, while the Huawei Honor V6 was used to display experimental stimulus materials.

### 2.2. Experimental design

**Figure 2:**
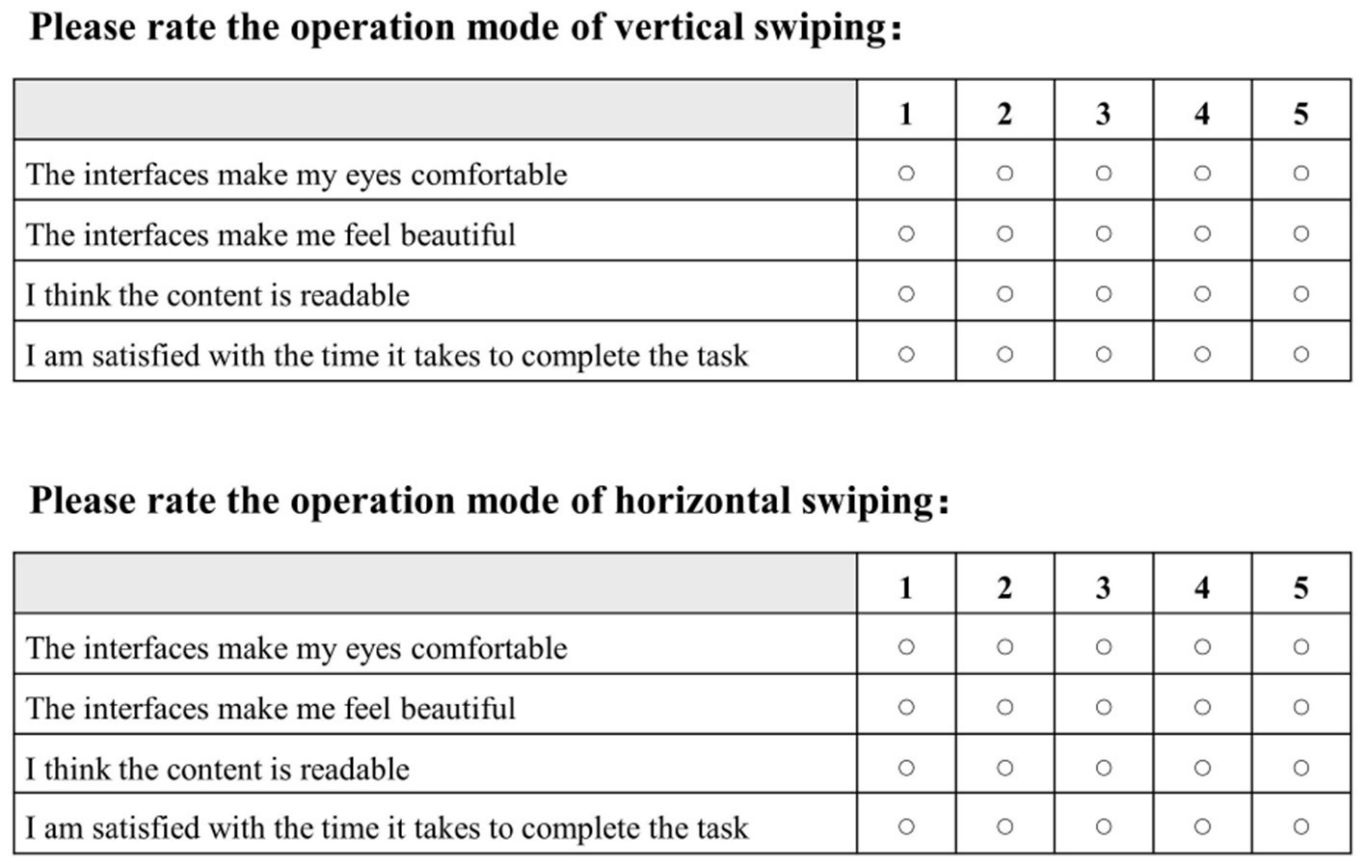
The subjective preference questionnaire used in the experiment.

An experiment-specific smart home interface was designed according to the purpose of the experiment. A total of one instruction page was included as well as six test pages. The instruction page introduced the experiment as a layout and swiping test and asked the participants to click on the operations as requested by the facilitator. The test pages had ten functional button modules with text on the buttons that are all commonly used function words for the smart home. The module at the top of the interface was labeled with a specific task and the participants were asked to swipe to find the specified function button and perform a single click. The test pages came in two different orders. In the first, the last three test pages were laid out horizontally whereas the first three test pages were arranged vertically. Second, the last three test pages were laid out vertically whereas the first three were arranged horizontally. Participants would be randomly selected to experiment in one order so that the effect of the order of operation on the participants could be excluded. When the test pages were laid out vertically, participants would be asked to find the specified function button by swiping vertically. When the test pages were laid out horizontally, participants had to swipe horizontally to find the specified function button. During the test, the participants were required to complete the task correctly to proceed to the next task, until the six test tasks were completed. After the experiment, the participants were asked to complete the subjective preference questionnaire, as shown in Figure 2. The participants needed to give authentic self-report feedback on the experimental process.

The interface was designed with a uniformly rounded square button shape and a gray background for all buttons except the question button, which was red for emphasis, to minimize the effect of color on the results of the experiment. The size of the buttons in all three interfaces in the same swiping direction was kept the same, taking into account the layout, the length of the vertical and horizontal swiping buttons was different and the width was the same.

The independent variable for this experiment was the two different swiping directions (vertical and horizontal) on the smart home interface, and the dependent variable was divided into three parts: first, the EEG data collected in real-time during the experiment, second, the eye-tracking data collected in real-time during the experiment, and third, the questionnaire data collected at the end of the experiment regarding the participants' emotional preferences as users. Another set of independent variables, namely gender, could be introduced in the later data analysis.

### 2.3. Procedures

First, the participants were taken to the experimental site at Nanjing Forestry University. Before the experiment, the participants were informed of the specific experiment and were allowed to stop the experiment at any time if they felt unwell. After receiving consent from the participants, user profiles were created and information such as age, gender, physical condition, and duration of the experiment were recorded.

**Figure 3:**
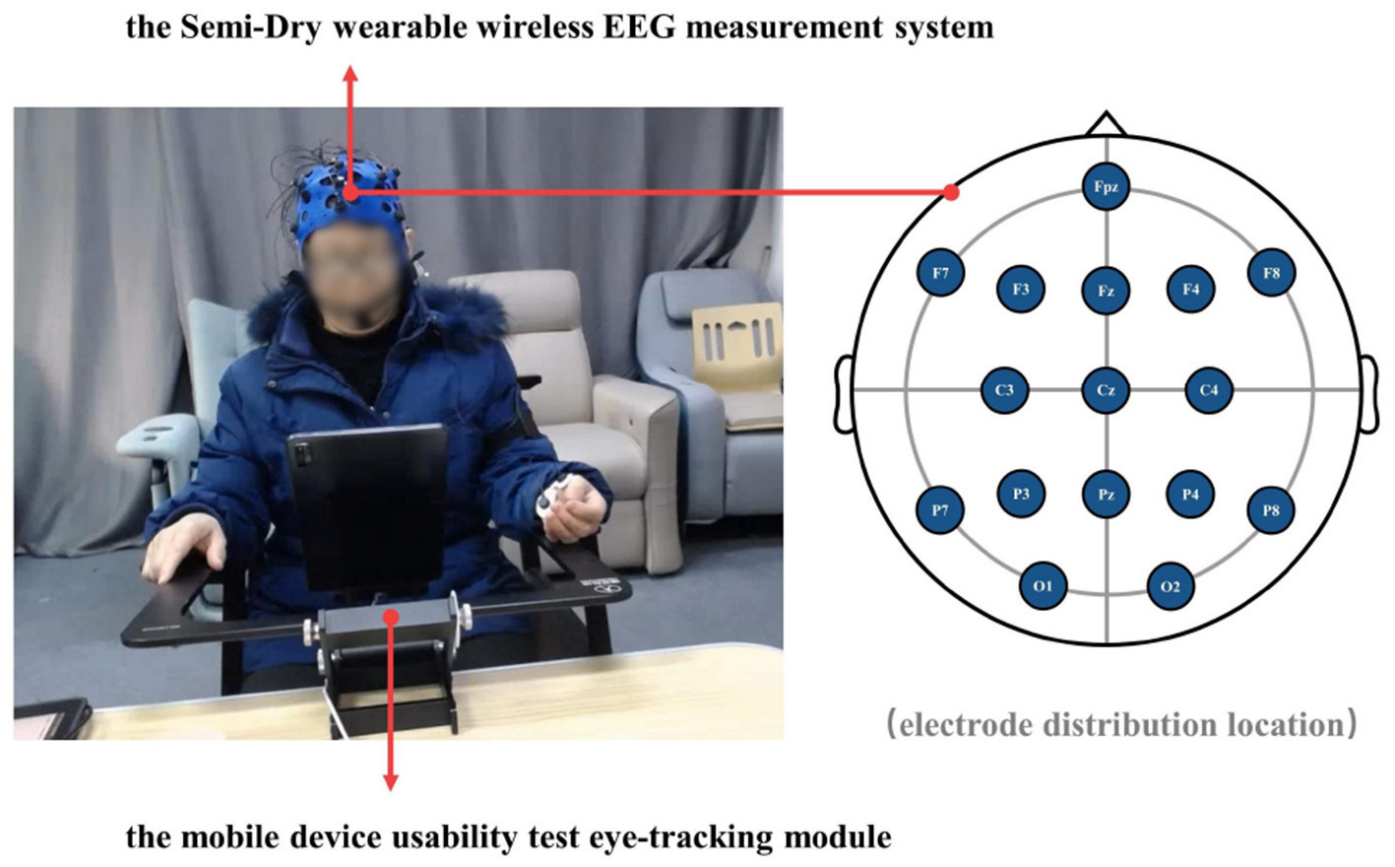
The experimental equipment and the distribution of the electrodes used in the experiments.

The participants then wore an EEG cap and were calibrated until all electrode points turn from red to green to ensure that the physiological data can be collected properly. The eye-tracking equipment was then commissioned to calibrate the participants' eye data and to ensure that the participants achieve a height of around 60–70cm. The setting effect of the Semi-Dry wearable wireless EEG measurement system and the mobile device usability test eye-tracking module is shown in Figure 3. This would be followed by an instructional phase where the facilitator informs the participants what is required and introduces the precautions to be taken. Before the experiment began, the participants were asked to rest with their eyes closed for 3 min. The facilitator then verbally told the participants the name of the function button they need to find and prompted them to click the button as fast and accurately as possible. The participants were asked to complete six interface tasks in sequence. At the end of the experiment, the participants were asked to fill in a subjective preference questionnaire, which would also be used for subsequent analysis.

### 2.4. Data acquisition and analysis

**Figure 4:**
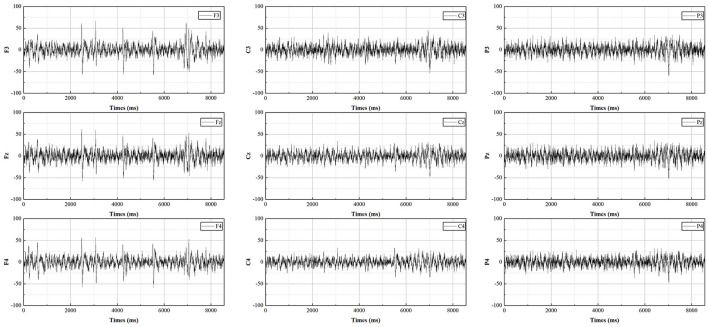
The average potential value for males during vertical swiping.

**Figure 5:**
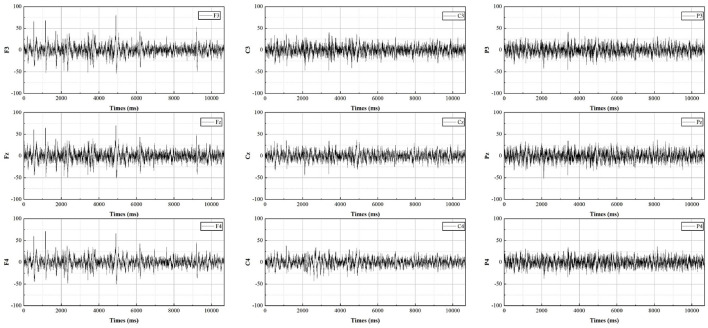
The average potential value for males during horizontal swiping.

**Figure 6:**
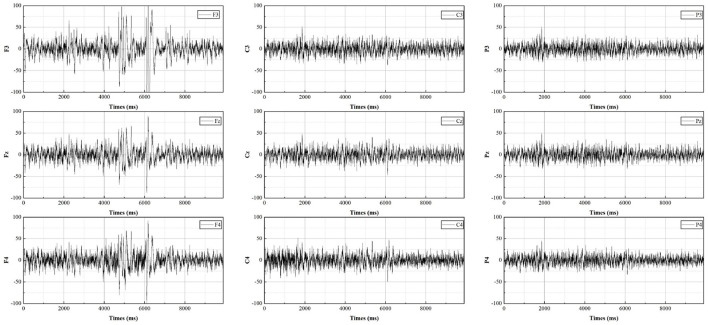
The average potential value for females during vertical swiping.

**Figure 7:**
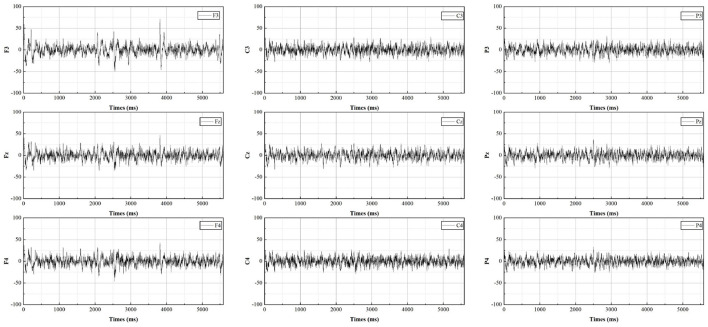
The average potential value for females during horizontal swiping.

**Figure 8:**
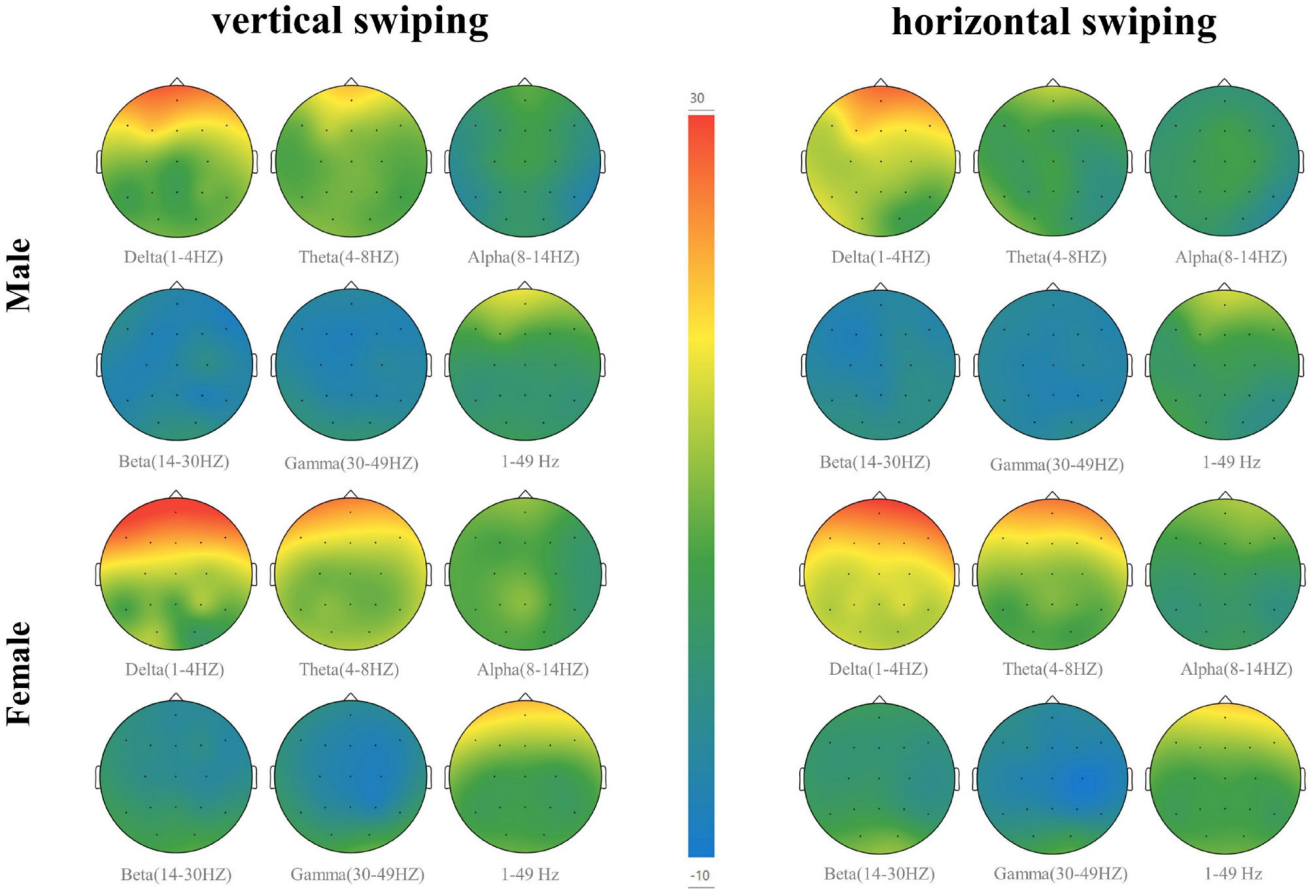
EEG topography based on different frequency bands. The mean power changes of participants in each band during vertical versus horizontal swiping were depicted.

The EEG measurements consist of sixteen electrodes, according to the international 10-20 electrode placement protocol for Fpz, F7, F3, Fz, F4, F8, C3, Cz, C4, P7, P3, Pz, P4, P8, O1, O2, distributed as shown in Figure 3. As most of the cognition-related analyses were concentrated in the prefrontal (F3, Fz, F4), central (C3, Cz, C4), and parietal (P3, Pz, P4) regions (Sun and Jin, 2021), these nine electrodes were selected for subsequent data analysis. The EEG data from all participants were averaged by electrode according to gender and swiping direction, allowing four sets of waveforms to be plotted, as shown in Figures 4–7. The changes in waveform amplitude were compared across the nine electrodes by swiping direction and gender, and repeated measures ANOVA was performed. A Mauchly sphericity test was first performed and as shown in Table 1, the test result of significance (*P* = 0.043 <0.05) did not satisfy the assumption of a spherical distribution and therefore a multivariate ANOVA was performed. Afterward, EEG topography based on the different frequency bands was plotted, as shown in Figure 8. The graph depicts the average power changes in each band as the participants performed vertical versus horizontal swiping, and distinguishes between the sexes of the participants. This map provides a visual representation of the EEG power changes in both swiping directions and can assist in later analysis. Next, fixation duration and pupil diameter were selected as eye-tracking metrics and a two-way ANOVA was performed separately. In addition, an ANOVA was performed on the subjective preference questionnaire to investigate the variability of the four descriptions in the questionnaire and to obtain participants' comfort preferences for the swiping direction as well as usability preferences by calculating the scores for each description. All data were analyzed using SPSS 26 and the significance level for all statistical tests was set at *p* <0.05.

**Table 1:** Mauchly's test of sphericity.

**Within subjects effect**	**Mauchly's W**	**Approx. Chi-Square**	**df**	**Sig**.	***Epsilonb*** ^*****b*****^		
					**Greenhouse -Geisser**	**Huynh-Feldt**	**Lower-bound**
**Electrodes**	0.001	53.986	35	0.043	0.383	0.736	0.125

^*b*^Indicates the degrees of freedom that can be used to adjust the significance mean test.

## 3. Results

### 3.1. EEG statistical results

**Figure 9:**
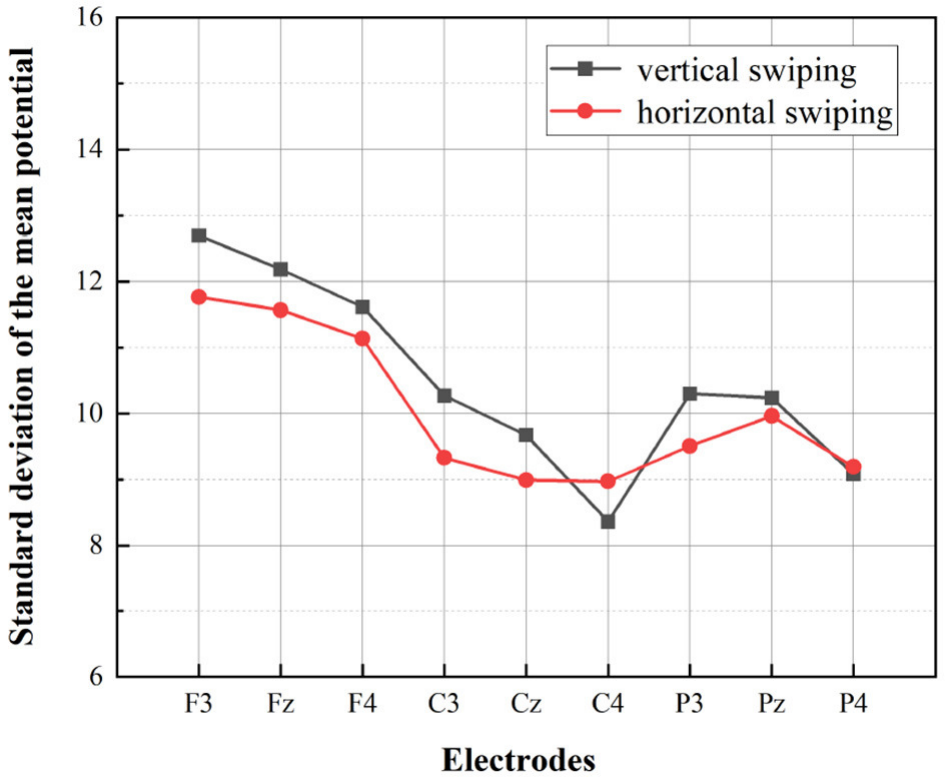
Statistics for males in two swiping directions. The standard deviation of the mean potential at the different electrodes for the two swiping directions are depicted.

**Figure 10:**
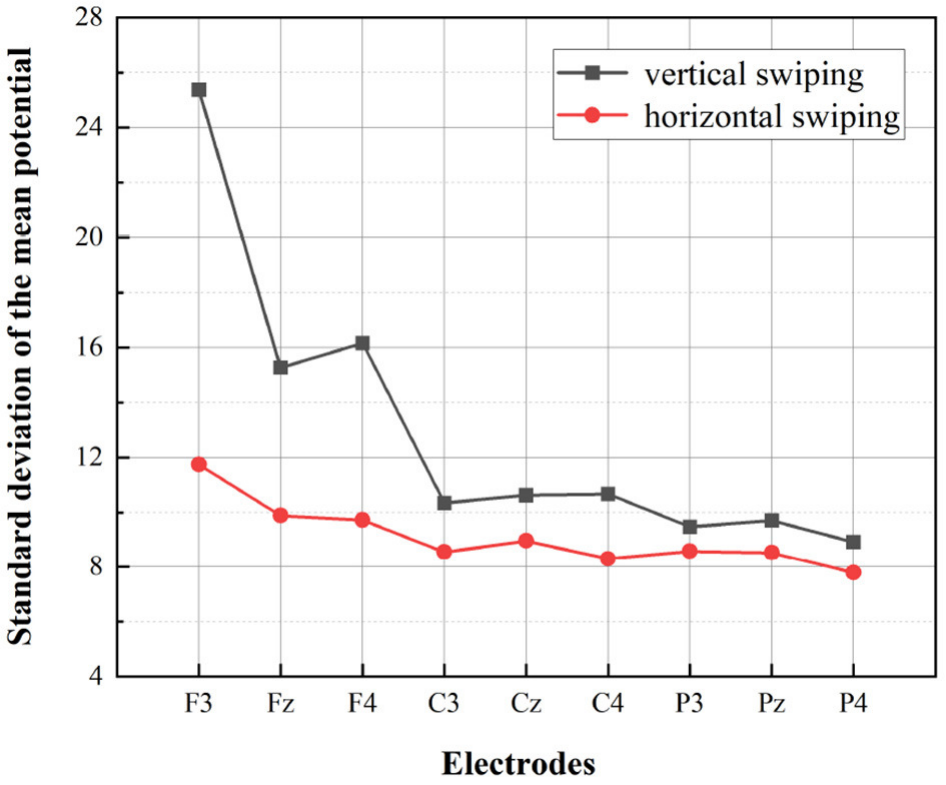
Statistics for females in two swiping directions. The standard deviation of the mean potential at the different electrodes for the two swiping directions are depicted.

A multivariate ANOVA was performed with gender, swiping directions, and electrodes as independent variables and mean potential value and standard deviation as dependent variables, as shown in Table 2. According to the results, the effect of swiping directions on the dependent variable showed a strong significance (*p* = 0.001 <0.05). This means that the use of different swiping directions for information retrieval in the interface leads to significantly different results for the mean potential value and the standard deviation of the potential value. According to Figure 9 as well as Figure 10, it is evident that vertical swiping causes a larger standard deviation of the mean potential compared to horizontal swiping, which means that the potential values of each electrode are more discrete when swiping vertically. And according to the statistical results, the effect of gender on the dependent variable is not significant (p = 0.085>0.05) and the potential values do not differ by gender. However, as can be seen in Figures 9, 10, the difference in swiping directions is relatively more pronounced for females.

**Table 2:** Results of multivariate analysis of variance.

**Effect**		**Value**	**F**	**Hypothesis df**	**Error df**	**Sig**.
Swiping Direction	Pillai's Trace	0.142	7.352^*b*^	2.000	89.000	0.001
	Wilk's Lambda	0.858	7.352^*b*^	2.000	89.000	0.001
	Hotelling's Trace	0.165	7.352^*b*^	2.000	89.000	0.001
	Roy's Largest Root	0.165	7.352^*b*^	2.000	89.000	0.001
Gender	Pillai's Trace	0.054	2.537^*b*^	2.000	89.000	0.085
	Wilk's Lambda	0.946	2.537^*b*^	2.000	89.000	0.085
	Hotelling's Trace	0.057	2.537^*b*^	2.000	89.000	0.085
	Roy's Largest Root	0.057	2.537^*b*^	2.000	89.000	0.085

^*b*^Indicates the exact statistic.

### 3.2. Eye-tracking metric statistical results

**Figure 11:**
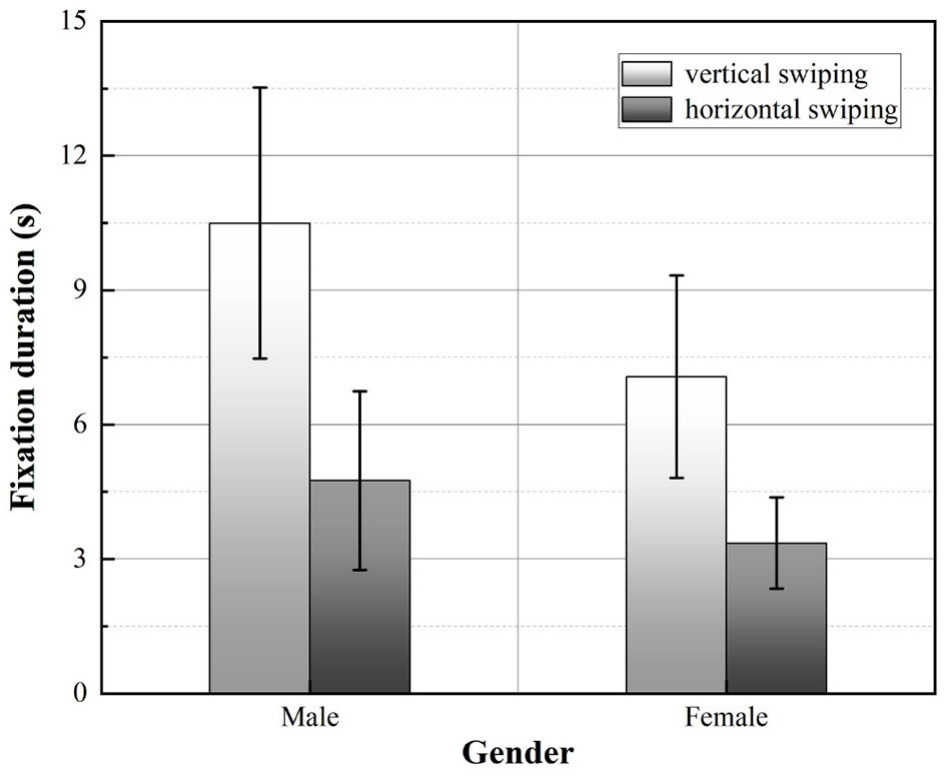
Comparison of the fixation duration averaged across participants. Error bars show the standard deviation values.

First, a two-way ANOVA of the mean fixation duration of the participants in the obtained experiments revealed a significant main effect of swiping direction (*F* = 6.242, *p* = 0.047<0.05), while the main effect of gender was not significant (*F* = 1.624, *p* = 0.250>0.05). According to the graph of the relationship between swiping direction and gender and fixation duration, as shown in Figure 11, it can be seen that the mean fixation duration was significantly longer for participants when swiping vertically compared to swiping horizontally, with males showing a more significant difference. In addition, male participants had a longer fixation duration than female participants, regardless of the direction of swiping.

**Figure 12:**
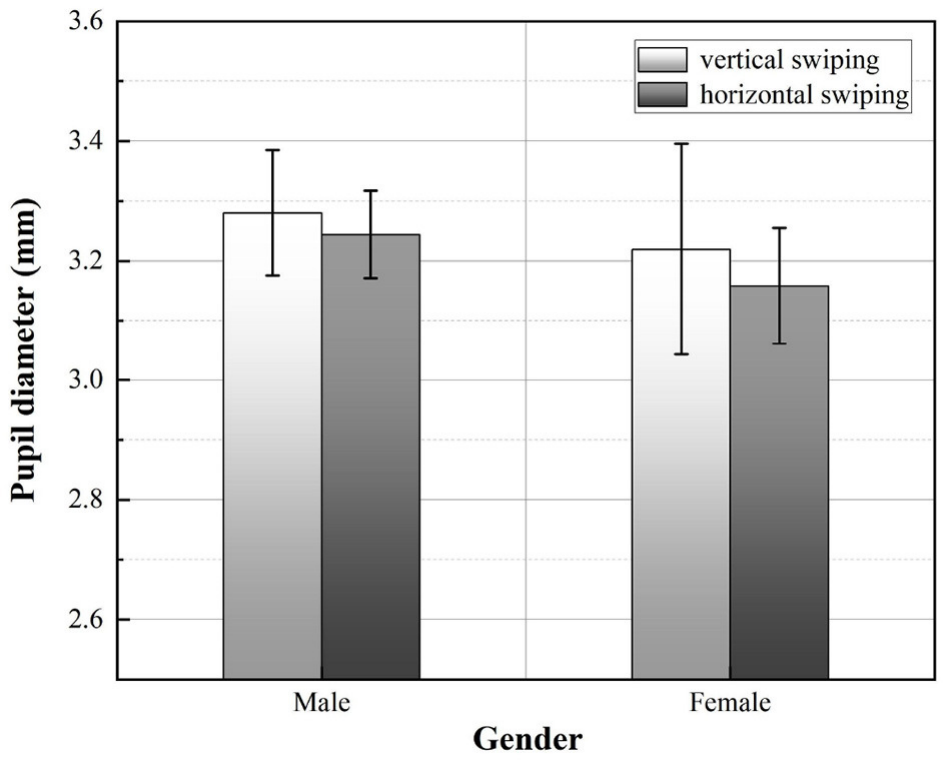
Comparison of the pupil diameter averaged across participants. Error bars show the standard deviation values.

Afterward, a two-way ANOVA was performed on the mean pupil diameter of the participants in the experiment obtained, and it was found that the main effect of swiping direction was not significant (*F* = 0.328, *p* = 0.576>0.05), nor was the main effect of gender (*F* = 0.846, *p* = 0.373>0.05). According to the graph of the relationship between swiping direction and gender and pupil diameter, as shown in Figure 12, it can be seen that the mean pupil diameter of participants did not differ significantly for different swiping directions. Compared to horizontal swiping, the mean pupil diameter was slightly larger than vertical swiping. The difference in mean pupil diameter between men and women was also not significant. Male participants had a slightly larger mean pupil diameter compared to female participants.

### 3.3. Statistical results of the subjective preference questionnaire

Analysis of variance (ANOVA) was used to investigate the variability of the four descriptors in the subjective preference questionnaire. According to the statistical results, the different swiping directions did not show any significance for “The interfaces make me feel beautiful” (*P* = 0.133 >0.05) and “I think the content is readable” (*P* = 0.34 >0.05). The results imply that there is no significant difference between the different swiping directions in terms of aesthetic and readability performance. In contrast, different swiping directions showed significant effects on "The interfaces make my eyes comfortable" (*F* = 14.520, *P* = 0.001 <0.05) and “I am satisfied with the time it takes to complete the task” (*F* = 4.909, *P* = 0.036 <0.05). This means that the different swiping directions have significant differences in comfort and satisfaction with time spent. Specifically, the mean score for the vertical swiping type (5.0 ± 0.00) was significantly higher than the mean score for horizontal swiping (4.15 ± 0.80) for the description “The interfaces make my eyes comfortable”. For the description “I am satisfied with the time it takes to complete the task”, the mean score for the vertical swiping type (4.85 ± 0.38) was also significantly higher than the mean score for the horizontal swiping type (4.38±0.65).

## 4. Discussion

### 4.1. Analysis of statistical results

The statistical results of the EEG above demonstrate that swiping direction has a significant effect on individual electrode potential values (*P* = 0.001<0.05) and that vertical swiping causes a greater standard deviation of the mean potential. Furthermore, according to Figure 8, it is evident that the mean power in the δ and θ bands were higher during vertical swiping compared to horizontal swiping, and the mean power in each band was higher in females compared to males. It has been shown that enhanced δ and θ bands exhibit more active task processing, implying better cognitive performance (Kober et al., 2016). In Ding et al. (2020), experimental results demonstrated that intelligent interfaces with higher user experience scores evoked stronger relative power in α, δ, and γ. And in Huang et al. (2019), experimental results showed that positive emotions tend to evoke higher average power. Thus, enhanced mean power in the δ and θ bands during vertical swiping represents more active cognitive processing, cognitive performance, and more positive emotions, which is consistent with the findings of the user subjective preference questionnaire (participants generally preferred vertical swiping and rated it highly). And the difference in mean power between males and females across the bands, especially in the δ and θ bands, shown in the figure, validates the conclusion of some previous experiments that δ and θ power are higher in females than in males when performing different cognitive tasks (Güntekin and Başar, 2007; Kober and Neuper, 2011). Through the analysis of EEG data, the present experiment demonstrates a significant effect of swiping direction on EEG data, with participants' greater preference for vertical swiping able to elicit greater standard deviation of mean potential values and elicit higher mean power in the δ and θ bands. There is no difference in the preference for swiping direction between males and females, but there are differences in EEG performance, with the cognitive task being more stimulating for females than for males.

The eye-tracking metrics used in this experiment, namely fixation duration (the total amount of time the participant's eye spends in an interface area), and pupil diameter, reflect the participant's emotional and cognitive state (Sari et al., 2015), including the participant's level of interest in the stimulus, emotional arousal, and mental load. Longer fixation duration indicates difficulty in extracting information or greater attractiveness (Ehmke and Wilson, 2007). Combined with the results of the subjective preference questionnaire (participants generally preferred vertical swiping and rated it highly), it can be assumed that the length of fixation during vertical swiping reflects the participants' arousal and the attractiveness of the interface task, rather than implying excessive mental load. Changes in pupil diameter can also reflect individual cognitive processes in response to input stimuli (Goldinger and Papesh, 2012), and cognitive load theory suggests that pupil changes are a direct reflection of increases or decreases in cognitive load (van der Wel and van Steenbergen, 2018), but scholars have shown that emotion also has an effect on pupil size and is not entirely representative of cognitive effort (Granholm and Steinhauer, 2004). When participants gazed at a target of interest, the pupil dilated, reflecting their arousal to the target's appearance. Although the direction of swiping did not have a significant effect on pupil diameter in this experiment, the mean pupil diameter of participants swiping vertically was slightly higher than swiping horizontally, which is consistent with the results of the fixation duration index.

### 4.2. Preference of the elderly for swiping direction

The results of the experiment showed that the participants all preferred vertical swiping, regardless of gender, which is a deviation from previous studies on swiping direction preference (Warr and Chi, 2013; Kim et al., 2016; Jeong and Liu, 2017; Fierrez et al., 2018). The results of this experiment suggest that previous findings appear to overstate the benefits of horizontal swiping, especially for simpler and more consistent information retrieval tasks. The results of this experiment go some way to validating the findings in Burnett et al. (2013), that vertical swiping makes people inclined to process information quickly (this experiment suggested that participants need to hit keys as fast and accurately as possible) and is more suitable for coherent information processing (the text retrieval task in this experiment was a coherent information processing task in favor of coherence). The experimental results also demonstrate that the user's preference for swipe direction is not constant, but depends to some extent on the interaction task the user is faced with. For example, in Yu et al. (2022a), the authors investigated swipe direction preferences for the function of adjusting temperature, a task in which horizontal swiping showed a stronger interaction advantage.

Furthermore, the experimental results cannot be excluded from being related to the usual smart device usage habits of the elderly. Through the user study, it was found that the participants all used touchscreen phones with the Android operating system, covering national brands such as Huawei, Oppo, and Xiaomi. The five most commonly used applications were instant messaging software (e.g., WeChat), text reading software (e.g. browser, news app, etc.), camera, video software, and online shopping software. Among this commonly used software, especially when reading the information, vertical swiping is still the preferred method of interaction. When the elderly are used to performing vertical swiping gestures, they become more accustomed to using vertical swiping gestures when exposed to a completely new interface (all participants showed that they had never been exposed to a smart home interface before), rather than choosing the horizontal swiping method for theoretical reasons due to line-of-sight processing (Rayner, 1998).

### 4.3. Smart home interface design for the elderly

The discrepancy between the experimental results in this paper and those of previous studies could suggest that the consideration of the elderly's needs for interfaces cannot rely on universal findings. When designing elderly-oriented smart home interfaces, an in-depth understanding of the elderly's preferences is required. Smart home interfaces are unfamiliar technologies to the elderly, and only when they are closer to their habits will they be less intimidated and resistant. In conjunction with the above analysis, the choice of interface interaction method needs to be made on a case-by-case basis, rather than uniformly using a theoretically more interactive method. In the case of the swipe direction studied in this paper, for example, vertical swipe gestures tend to be consistent, while horizontal swipe gestures tend to be more about switching and segmentation, so the direction of the interface needs to be designed according to the specific function.

### 4.4. Limitations

There are still some limitations to this experiment. Firstly, the task set-up in this paper is relatively simple and does not require complex information processing, so it may not fully present the differences between horizontal and vertical swiping at a cognitive level. In practice, users would need to perform more complex sequential operations, which, together with the influence of icons, etc., may lead to differences in the results. Secondly, the experiment was conducted with the participants in a smoother sitting position. In a real-world scenario, where the user may be standing or walking, it is worth considering whether these postures have an impact on the processing of the line of sight and therefore on the performance of the interaction. Thirdly, differences in screen resolution, pixel size, visual performance, sensitivity, etc. of the interface used in practice also affect the user's interaction performance (Huang and Menozzi, 2014; Hancock et al., 2015; Yamazaki and Watanabe, 2017; Zhou and Xu, 2022). Fourthly, although none of the participants in this experiment had experience with smart home interfaces, their familiarity with smart devices may have influenced the results to some extent. There may also be potentially subtle differences between iPhone users and Android device users. These limitations are all areas that could be investigated in depth.

In addition, all three research instruments involved in this paper have limitations. The subjective preference questionnaire is the easiest to obtain, but it does not allow for simultaneous tracking of subjects' responses, i.e., it does not allow for immediate feedback. In addition, subjects are not always able to express their thoughts and ideas truthfully and accurately, so accuracy cannot be guaranteed. Eye-tracking data itself reflects behavior and does not directly reflect cognitive and thinking processes. The interpretation of the data results is not uniform and needs to be determined in conjunction with other data results. In contrast, EEG data is more objective and significant and is an effective way to study user preferences. However, there are some drawbacks: the data results of EEG may be affected by environmental factors, and the test scenarios may not reflect the real usage scenarios. Nevertheless, this paper uses three research tools at the same time, and to a certain extent, the results of user preferences are considered comprehensively and have high credibility.

## 5. Conclusion

This paper focuses on older persons and studies their swiping direction preference. The participants' preferences for the swiping direction were obtained through EEG data and subjective preference questionnaire data. The results showed that regardless of gender, participants preferred vertical swiping. The EEG data showed that vertical swiping caused greater mean potential deviation than horizontal swiping and enhanced mean power in the δ and θ bands. This indicates more active task processing, superior cognitive performance, and a more positive mood. Eye-tracking metrics data showed that participants fixed longer and had slightly larger pupil diameters when swiping vertically, suggesting that vertical swiping was more arousing and engaging. These objective data results are consistent with the results of the subjective preference questionnaire data. The experimental results in this paper prove the interactive advantage of vertical swiping in simple and coherent information processing tasks to some extent, but it is not excluded that it is affected by the participants' usual use habits. In a follow-up study, we can more deeply study the influence of the elderly's use habits on the swiping direction preference of the smart home interface. In addition to the swiping direction, there are many design elements of the smart home interface that need to be explored in-depth, including icon size, icon style, color matching, etc. Only by truly considering the needs and preferences of the elderly, can we design a smart home interface that is accepted by older persons, to truly improve the home environment of the elderly and enable them to have the ability to live independently and comfortably.

## Data availability statement

The original contributions presented in the study are included in the article/supplementary material, further inquiries can be directed to the corresponding author.

## Ethics statement

The studies involving human participants were reviewed and approved by Ethics Committee of Nanjing Forestry University. The patients/participants provided their written informed consent to participate in this study.

## Author contributions

All authors listed have made a substantial, direct, and intellectual contribution to the work and approved it for publication.
